# Eosinophilic Asthma Secondary to Adjuvant Anti-PD-1 Immune Checkpoint Inhibitor Treatment in a Melanoma Patient

**DOI:** 10.1155/2022/2658136

**Published:** 2022-04-30

**Authors:** P. Kissoonsingh, B. Sutton, Syed U. Iqbal, Lalit Pallan, Neil Steven, L. Khoja

**Affiliations:** ^1^College of Medical and Dental Sciences, University of Birmingham, Edgbaston, Birmingham B15 2TT, UK; ^2^University Hospitals Birmingham NHS Foundation Trust, Queen Elizabeth Hospital, Department of Oncology, Birmingham B15 2TH, UK; ^3^Institute of Immunology and Immunotherapy, College of Medical and Dental Sciences, University of Birmingham, Edgbaston, Birmingham B15 2TT, UK

## Abstract

**Background:**

Adjuvant immune checkpoint inhibitors are a new standard of care in melanoma. However, the immune related toxicity associated with these agents can be serious, and the long-term implications are yet to be defined especially in the adjuvant setting. We report, to our knowledge, the first case of anti-PD-1-induced eosinophilic asthma in a melanoma patient treated with adjuvant pembrolizumab. *Case Presentation.* A 72-year-old man commenced pembrolizumab in the adjuvant setting after resection of a stage IIIB cutaneous melanoma. The patient experienced episodes of breathlessness 4 weeks after cycle 1. These episodes were nocturnal and caused acute respiratory distress and cough, occasionally waking him up. The episodes progressed, and he was admitted after cycle 2 with a productive cough, wheeze, and breathlessness. Observations showed saturations on air of 94% and a respiratory rate of 19/min. The only laboratory abnormality was a raised eosinophil count of 1.1 × 10^9^. Spirometry showed a FEV1 of 2.57 (91% predicted), FVC of 4.04 (108% predicted), and ratio of 64%. Peak expiratory flow rate was 94% predicted, and corrected gas transfer was 6.29 (78% predicted) with KCO 1.18 (93% predicted). FeNO was raised at 129 indicating inflammation of his airways, and peak flow was 422 l/min. CT of the chest did not show pneumonitis or other lung pathology. A diagnosis of acute eosinophilic asthma was made. Treatment with steroids and beclometasone dipropionate and formoterol inhaler produced rapid resolution of symptoms and normalisation of the eosinophil count. Pembrolizumab was safely recommenced once steroids had discontinued and symptoms had resolved.

**Conclusions:**

Specialist respiratory input was needed for optimal patient management and is ongoing. Although a safe rechallenge with pembrolizumab was possible, treatment in the adjuvant setting is curative in intent and long-term safety follow-up is required to assess for delayed toxicity and long-term health implications. This is likely to require large regional/national/international databases to detect, monitor, and educate the wider medical community as these patients are followed up in primary care following initial specialist follow-up.

## 1. Background

Anti-PD-1 immune checkpoint inhibitor therapy is approved for melanoma in the adjuvant (curative) setting after surgery [1, 2]. These agents alone and in combination with ipilimumab (anti-CTLA-4 inhibitor) were first approved in metastatic melanoma [3, 4], and their toxicity profiles are well characterised in this setting [5, 6]. The majority of these toxicities resolve albeit with significant management in some instances. However, some toxicity requires lifelong intervention. The longer-term implications of these toxicities and any further longer term health implications are unknown.

In the adjuvant setting, toxicity has reflected that of the metastatic setting [7], but there is as yet a paucity of data on long-term outcomes in terms of both toxicity and any subsequent health issues and overall survival outcomes from melanoma. Moreover, the risk benefit ratio in the adjuvant setting differs widely from that of metastatic disease.

Here, to our knowledge, we report the first case of anti-PD-1-induced eosinophilic asthma in the adjuvant setting, in a melanoma patient treated with pembrolizumab. We discuss the management and possible implications for the patient.

## 2. Case History

A 72-year-old male was referred for adjuvant therapy following resection of a stage IIIb malignant melanoma. The patient first presented 2 years earlier with a midscalp lesion; a punch biopsy of which showed an amelanotic SOX10 and S100 positive melanoma (Breslow thickness unknown). The lesion had completely regressed by the time of referral for definitive treatment, and wide local excision did not show malignancy. A year later, a skin lesion from the right supra-auricular skin/occipital scalp was resected. This proved to be a 13 mm subcutaneous nodule consistent with metastatic melanoma. Histology revealed pleomorphic epithelioid and spindle-shaped cells with mitotic figures, positive for S100 protein and SOX10. Perineural invasion was seen. A CT scan of the body did not show metastatic disease. Staging was Tx N2c M0 stage III. The patient had a background of hypertension, previous MI, and coronary stent. There was no history of autoimmune or inflammatory conditions. There was no history of chronic obstructive airway disease, but he was an ex-cigar smoker having stopped 32 years ago. His medication consisted of atorvastatin 20 mg, latanoprost, lisinopril 10 mg, and rivaroxaban 20 mg. Adjuvant 6 weekly pembrolizumab was commenced 6 weeks from surgery and planned to continue for one year in total.

The patient started to have episodes of breathlessness 4 weeks after cycle 1/first dose of pembrolizumab. These episodes tended to be at night and caused acute respiratory distress and cough, occasionally waking him up at night. He had some exposure to dust during this period as a result of domestic building work. The episodes become more severe and prolonged, and he was admitted after cycle 2 of pembrolizumab for further investigation. At that time, he had a productive cough, wheeze, and breathlessness.

**Figure 1:**
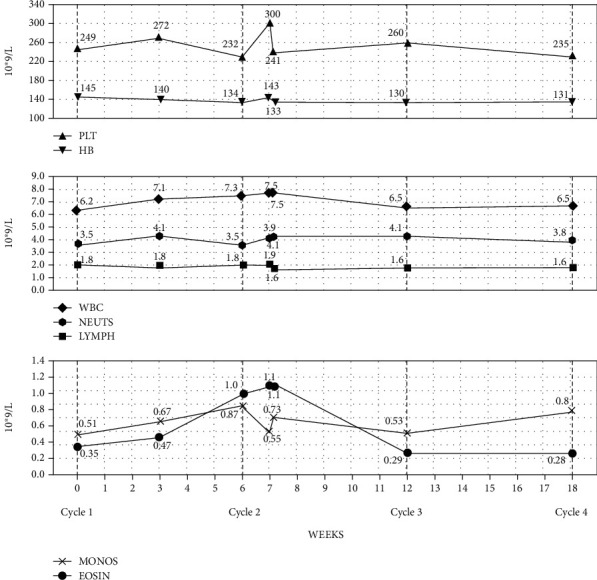
Line graphs depicting serial full blood count values over time over cycles 1-4. Eosinophils were the only subset of cells that elevated above normal range after cycle 1 before returning to normal at time of cycle 3. Normal ranges for haemoglobin (130-162), white cell count (4.3-11.2), platelets (150-400), neutrophils (1.90-7.90), basophils (0.05-0.10), monocytes (0.30-0.90), eosinophils (0.03-0.44), and lymphocytes (0.50-4.00).

**Figure 2:**
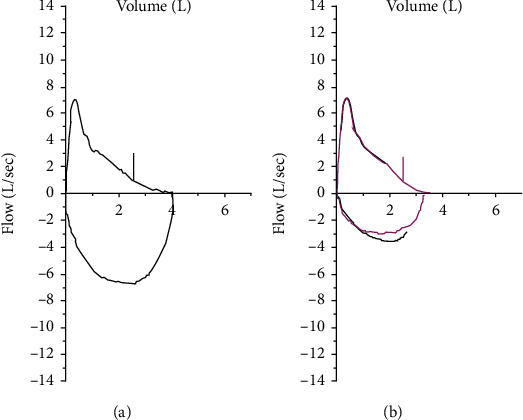
(a) Spirometry during acute admission with cough, wheeze, and shortness of breath. Spirometry indicates obstructive airways with raised FeNO supporting a process of inflammation in the airways. (b) Spirometry 6 months after acute episode requiring admission, values now normal including the FeNo.

On admission, his saturations were 94% on air and his respiratory rate was 19. Blood tests showed normal renal and liver function, a CRP count of 3 mg/l, and a full blood count that revealed an isolated raised eosinophil count of 1.1 × 10^9^. There was no evident rash, and the patient had not received any other new medications (excluding a possible diagnosis of DRESS (drug-related eosinophilia with systemic symptoms)). On review of serial blood tests, an isolated raised eosinophil count of 0.47 × 10^9^ first occurred 3 weeks after cycle 1 pembrolizumab and peaked at 1.1 × 10^9^ coinciding with his admission postcycle 2 of treatment (Figures 1(a) and 1(b)) (autoantibody screen was not performed either pre- or on immunotherapy). Spirometry during admission showed a FEV1 2.57 (91% predicted), FVC 4.04 (108% predicted), ratio of 64% (peak expiratory flow rate 94% predicted), gas transfer corrected 6.29 (78% predicted), and KCO 1.18 (93% predicted) (Figure 2(a)). He also had a raised FeNO of 129 indicating inflammation of his airways and a peak flow of 422 l/min. CT of the chest did not show pneumonitis or other lung pathology.

An immune adverse event was suspected in this patient. Pneumonitis secondary to immune check point inhibitor therapy would be the most common respiratory toxicity and was the primary differential diagnosis, but the CT chest was clear. Immune-related myocarditis or pericarditis could have been less likely possibilities, but there the clinical signs were not consistent with these. The initial intermittent nature of the breathlessness and the nocturnal timing of it, along with the history of dust exposure and progressive worsening of symptoms, made asthma the most likely diagnosis. The raised eosinophil counts over time added weight to an allergic and inflammatory process being the cause. A diagnosis of acute eosinophilic asthma was made, and treatment instigated with 40 mg prednisolone for 5 days and fostair (beclometasone dipropionate and formoterol) 100/6 two puffs twice daily. There was a rapid response to treatment with improvement in all of his symptoms. The eosinophil count declined to a normal range within 2 days of prednisolone treatment, and peak flow improved to 590 l/min indicating reversibility of the acute obstruction. Repeat spirometry 6 months later showed FEV1 2.61 (89% predicted), FVC 3.65 (95% predicted), ratio of 93% (peak expiratory flow rate 97% predicted), and FeNo 17 ppb (Figure 2(b)).

Pembrolizumab was recommenced once steroids had discontinued and symptoms had resolved, on schedule, 6 weeks after cycle 2. The patient has not required any further acute treatment for his asthma and continues on fostair (beclometasone dipropionate and formoterol) 200/6 and salbutamol as required (using them with a spacer). Peak expiratory flow rate remains stable at an average of 500 l/min (which is within normal range for his age). His night time breathlessness has completely resolved, and his exercise tolerance is back to his baseline. During follow-up to date, he has described some rhinitis, but this is mild and has not required further investigation or management.

## 3. Discussion

Respiratory toxicity with checkpoint inhibitors is well documented with a wide range of toxicities reported including dyspnoea, pneumonitis, pleural effusion, pulmonary sarcoidosis, acute fibrinous organizing pneumonia, eosinophilic pneumonia, adult respiratory distress syndrome, and lung cavitation [8, 9]. The most common is pneumonitis with a reported incidence of 1.7% of any grade in melanoma with single-agent anti-PD-1 agents [6]. Exacerbation of asthma has been reported and may be fatal [8, 10]. Predictive markers for immune checkpoint-related toxicity are lacking. Eosinophil counts have been proposed as a possible marker of increased inflammation and immune activation with subsequent improved survival outcomes [11–13] or increased risk of toxicity [14].

Asthma is characterised by inflamed hyperresponsive bronchial airways and reversible airflow obstruction [15]. It is increasingly recognised that it is a heterogenous disease with different pathophysiologic mechanisms driving airway inflammation and therefore subsequent differences in outcomes and effectiveness of treatments. Eosinophilic asthma represents approximately fifty percent of asthma with varying phenotypes [16]. It is frequently associated with “fixed” airflow obstruction, decreased FVC, and increased residual volume, with late (in life) onset and typical symptoms of dyspnoea on exertion rather than wheeze [15, 17]. Exacerbations can be severe with development of steroid reliance and then resistance over time. A typical comorbidity at presentation or one which subsequently develops is chronic rhinosinusitis with nasal polyposis. Diagnostic tests include demonstration of airflow variability, raised peripheral eosinophil counts, raised FeNo, and induced sputum testing with a typical finding of increased inflammatory cells and eosinophilia [15, 17].

Our patient had a typical presentation of eosinophilic asthma which occurred after the first dose of pembrolizumab. There was no history of preexisting respiratory conditions, and other diagnoses were excluded. The only possible causative agent was the pembrolizumab. The diagnosis was made based on the clinical presentation, the reversible peak expiratory flow rate readings before, during, and after treatment, and the spirometry which has also improved with treatment. Furthermore, the patient during the acute presentation had a high FeNo with evidence of variable airflow which also settled with treatment. These findings fit with NICE criteria for diagnosis of asthma. Treatment was rapidly successful in controlling his symptoms, and pembrolizumab rechallenge has been successful alongside regular inhaler use to control any asthma symptoms.

The patient is halfway through the planned year of pembrolizumab adjuvant therapy to date without further asthma exacerbations. We found one reported case in the literature of asthma induced by anti-PD-1 agent nivolumab in a stage IV lung patient. This patient had a very similar presentation and diagnostic findings as our patient and symptoms resolved with inhalers alone. Although not described as such, that case was consistent with eosinophilic asthma [18].

Treatment in the adjuvant setting is curative in intent, and long-term safety follow-up is required to assess for delayed toxicity and long-term health implications following adjuvant immune checkpoint inhibitor therapy. This is likely to require large regional/national/international databases to detect, monitor, and educate the wider medical community as these patients are followed up in primary care following initial specialist follow-up. Regarding our patient, subsequent follow-up postcompletion of the year's therapy will reveal if exacerbations of our patient's asthma develop and become problematic or indeed if he develops any other toxicity related to (pembrolizumab-induced) eosinophilia or pembrolizumab treatment in general. A multidisciplinary approach with respiratory expertise was needed in our patient, and this should be the approach in managing and following up patients postadjuvant immune checkpoint inhibitor treatment.

## 4. Patient Perspective

I first had a spot removed from my scalp in 2019. I was told it was most likely a melanoma that had spontaneously disappeared. In 2020, I noticed a lump behind my right ear which grew from a pea to almond sized. I was concerned as my surgery was delayed due to the COVID pandemic, but the lump was completely removed and shown to be a melanoma. I was told that the melanoma could recur, but if I had adjuvant treatment, meaning treatment to complement the surgery and prevent the melanoma from returning, the chances of this happening could be reduced. I opted to start pembrolizumab treatment given intravenously every 6 weeks for one year. It was explained that this was an immunotherapy meaning it uses the immune system to attack the melanoma cells. I was counselled about possible side effects which would be immune related as in an immune reaction in any part of my body.

After the first cycle of treatment, I started to have shortness of breath. This tended to be at night and made me feel unwell lasting a few hours. I attended accident and emergency and was told my CXR and oxygen saturations were fine and that I could take antihistamines as I may be having allergic reactions. I also consulted my GP who gave me an inhaler to use if I had another episode. The shortness of breath kept recurring. I had cycle 2 pembrolizumab, and the oncology doctor who assessed me beforehand told me that the drug may have sensitized me to allergens, and this could be causing the breathlessness. An opinion from the respiratory physicians would be sought. After cycle 2, I kept having the breathlessness and could not cope any more at home; I phoned the red card for oncology emergencies and was admitted to hospital for assessment. Several tests were done to assess my lungs; I had a CT scan which did not show any inflammation (a possible side effect of the pembrolizumab) and lung function tests after which a respiratory doctor also assessed me. High-dose steroids and an inhaler treatment stopped my symptoms completely. I was discharged home to finish the course of steroids but continue using the inhaler. The diagnosis for my breathlessness was found to be asthma. I was pleased to have a cause for the breathlessness found and for the treatment to have worked. I was assessed in clinic after discharge to see if I could continue with pembrolizumab. It was explained that there was a risk of the breathlessness returning if I restarted treatment, and this could either be treated in the same way again, or if the attack was too severe, then the treatment would have to be stopped. There was also a risk of other immune-related side effects if pembrolizumab restarted. I felt well and am keen to finish my treatment as I do not want the melanoma to recur, so I opted to restart. So far everything is going smoothly and I have had no further issues. I use my inhaler when needed but as time as gone on, I have used it less and more recently not at all. I have seen the specialist asthma nurses in clinic who are pleased with my progress and are going to repeat my lung function tests in a few months to compare to the ones done during my admission.

I discussed my side effect with the oncology doctor last time I was seen in clinic and asked about how long the pembrolizumab remains in the body. She explained that the half-life (time taken to reduce to half the amount) is ~30 days, and the time to completely clear the drug from the body from the last dose would be ~5 times this period. This means that the antibody is not cleared quickly and that toxicity can therefore happen late into treatment and even after treatment is stopped. I will have to have close monitoring for side effects for at least 6 months after finishing treatment, and I will have close monitoring by CT and assessment in person by a doctor to test if my melanoma returns in the years to come. My asthma may remain stable or it may worsen or recur. Treatment will be given to control it.

## Data Availability

Data are available on clinical records held in the hospital electronic database.
